# Prevalence and Impact of Zinc Deficiency on Clinical Outcomes in Inflammatory Bowel Disease

**DOI:** 10.3390/nu17213378

**Published:** 2025-10-28

**Authors:** Hend Almuhaya, Raghad Alsalamah, Asma Sallam, Amgad Alonazy, Atheer AlAwwad, Gamal Mohamed, Abdulelah Almutairdi, Mashary Attamimi, Badr Al-Bawardy

**Affiliations:** 1 Department of Internal Medicine, Division of Gastroenterology and Hepatology, King Faisal Specialist Hospital, Riyadh 11564, Saudi Arabia; 2 Department of Internal Medicine, King Faisal Specialist Hospital, Riyadh 11564, Saudi Arabia; 3 Department of Biostatistics, Epidemiology and Scientific Computing, King Faisal Specialist Hospital, Riyadh 11564, Saudi Arabia; 4 College of Medicine, Alfaisal University, Riyadh 11533, Saudi Arabia; 5 Department of Internal Medicine, Section of Digestive Diseases, Yale School of Medicine, New Haven, CT 06510, USA

**Keywords:** micronutrients, zinc, Crohn’s disease, ulcerative colitis, inflammatory bowel disease

## Abstract

**Background:** Inflammatory bowel disease (IBD) can lead to zinc deficiency, which plays a critical role in immune function and tissue repair. This study aims to determine the prevalence, clinical characteristics, and impact of zinc deficiency in IBD patients. **Methods:** This is a retrospective study of patients aged ≥14 years with confirmed IBD and available zinc level measurement. Zinc deficiency was defined as a level <10.6 µmol/L. Primary outcomes included the prevalence of zinc deficiency and the characterization of the clinical profile of patients with zinc deficiency. Secondary outcomes included IBD-related hospitalizations, emergency room visits, surgeries, and complications (anemia, small bowel obstruction, new perianal disease, intra-abdominal abscess). **Results:** Among 447 patients (54.4% male; 79.2% Crohn’s disease) with a median age of 29 years (IQR: 22–38), 45.6% had zinc deficiency (83.8% Crohn’s disease). Zinc-deficient patients had higher C-reactive protein and fecal calprotectin (both *p* < 0.001) levels, and were more likely to be on corticosteroids (*p* = 0.04). Zinc deficiency was associated with higher rates of IBD-related hospitalizations (48.0% vs. 17.7%), surgeries (19.6% vs. 5.8%), complications (30.4% vs. 12.4%), and emergency room visits (40.2% vs. 17.3%) (all *p* < 0.001). Upon multivariate analysis, predictors of IBD-related hospitalization were zinc deficiency (OR 2.42, 95% CI 1.07–5.48, *p* = 0.03) and low albumin (OR 9.03, 95% CI 3.38–24.15, *p* < 0.001). Zinc deficiency was associated with IBD-related surgeries (OR 5.23, 95% CI 1.27–21.45, *p* = 0.02) and complications (OR 3.98, 95% CI 1.52–10.41, *p* = 0.005). **Conclusions:** Zinc deficiency is prevalent in patients with IBD, associated with a high inflammatory burden, and was linked to worse clinical outcomes after controlling for markers of inflammation.

## 1. Introduction

Inflammatory bowel diseases (IBDs) such as Crohn’s disease (CD) and ulcerative colitis (UC) are chronic conditions characterized by intestinal inflammation and are associated with multiple complications [1]. Crohn’s disease can lead to complications such as strictures, fistula, and abscesses. Ulcerative colitis can also be progressive and lead to extension of inflammation, increased risk of colonic neoplasia, submucosal fibrosis, and reduced rectal compliance [2]. Malnutrition is an important complication of IBD. The prevalence of malnutrition in IBD has ranged globally from 6.1 to 92.5%, with no reported differences between CD and UC [3]. A multicenter European study reported disease-related malnutrition in 23% of patients with IBD (26.3% in CD and 19.7% in UC), compared to 49.5% in a large Chinese cohort (85.5% in CD and 47.6% in UC). In a single-center study from the United States, the prevalence of malnutrition was 14% (15% in CD and 12% in UC) [4,5,6]. The differences in malnutrition rates in different regions are likely impacted by multiple factors including socioeconomic, genetic, healthcare access, and disease-related factors [5].

Malnutrition in IBD is multifactorial and can be a result of increased energy requirements with chronic inflammation, malabsorption, decreased oral intake, and enteric nutrient loss [7]. Chronic intestinal inflammation in IBD causes structural and functional gut damage, impairing nutrient digestion and absorption. Persistent inflammation, driven by cytokines such as tumor necrosis factor and interleukins 1 and 6, promotes catabolism and anorexia, further worsening nutritional status [7]. The spectrum of malnutrition in IBD includes not only protein-calorie deficits but also micronutrient and trace element deficiencies. Zinc is one of the key trace elements which can frequently be deficient in IBD [3].

Zinc, a co-factor for numerous enzymes, plays a crucial role in multiple physiological functions including metabolism, cognitive function, reproductive function, cellular growth and development, and importantly, the immune system and wound healing [8]. Within the immune system, zinc plays a role in both humoral- and cell-mediated immunity, affecting neutrophils, T-cell and B-cell functions [9]. Therefore, investigating the status of zinc deficiency in IBD and its impact on outcomes is of interest. Zinc deficiency in IBD is relatively common and has been reported to occur in up to 50% of patients [3,10]. In patients with CD, zinc deficiency has been observed in up to 54% of individuals, while in UC, zinc deficiency has been reported in up to 41% of individuals [10]. The etiology of zinc deficiency in IBD may be related to impaired absorption, low dietary intake, diarrhea, high-output fistula, and glucocorticoids which can impair zinc absorption [10,11,12].

The impact of zinc deficiency on clinical disease course and outcomes remains partially investigated. Animal studies have demonstrated that zinc deficiency results in exacerbation of colitis in mice due to activation of the interleukin-23/TH17 axis [13]. Limited clinical studies have shown that zinc deficiency is associated with unfavorable outcomes in IBD patients. In a large cohort, CD patients with low serum zinc levels had approximately a 50% higher risk of hospitalization or disease-related complications, and were nearly twice as likely to require IBD-related surgery when compared to those with normal zinc levels. In UC, zinc deficiency was associated with approximately a twofold higher risk of hospitalization and demonstrated a trend towards significantly increased disease-related complications [14]. In another study, however, zinc levels in IBD were not associated with disease activity [15]. Therefore, the aim of this study is to examine the prevalence, clinical characteristics, and outcomes of zinc deficiency in a very well-characterized real-world cohort of patients with IBD.

## 2. Methods

### 2.1. Study Design

We performed a single-center retrospective study of patients aged ≥14 years with a confirmed diagnosis of IBD including CD, UC, IBD-unclassified (IBD-U), and pouchitis; patients included in the study had documented zinc levels from 1 January 2003–1 January 2024. The study protocol was approved by the Institutional Review Board. We collected demographic information at time of zinc level recording, including age, sex, smoking status, as well as disease-related variables including date of IBD diagnosis, disease location, phenotype, and presence of documented extraintestinal manifestations. Other variables included all current medical therapies for IBD; history of intestinal surgeries prior to zinc level measurement and laboratory results within 30 days of zinc level measurement, including baseline C-reactive protein (CRP); fecal calprotectin (FCP); and albumin. High CRP was defined as a level ≥ 5 mg/L and high FCP was defined as a level ≥ 250 μg/g [16,17]. Medical therapies were characterized as immunomodulators (azathioprine; mercaptopurine; methotrexate) or advanced therapies (infliximab; adalimumab; golimumab; certolizumab-pegol; vedolizumab; ustekinumab; upadacitinib; tofacitinib).

### 2.2. Outcomes

Zinc deficiency was defined as a level < 10.6 umol/L as per our institution’s assay and consistent with previously published studies evaluating zinc deficiency [18]. Non-fasting serum zinc levels were measured using Inductively Coupled Plasma Mass Spectrometry (ICP-MS), a highly specific and sensitive analytical technique used to determine patients’ zinc levels. ICP-MS uses high-temperature plasma to ionize elements, which are then detected based on mass-to-charge ratios, allowing detection at parts-per-billion (ppb) or even parts-per-trillion (ppt) levels. Outcomes were compared between patients who had a zinc deficiency at baseline and in those with normal zinc levels at baseline. The primary outcomes were the prevalence of zinc deficiency in IBD patients, and the characterization of the clinical profile of patients with zinc deficiency. Secondary outcomes included rates of IBD-related hospitalizations, emergency room (ER) visits, IBD-related surgeries, and IBD-related complications. IBD-related complications was a composite outcome of either anemia (defined as hemoglobin < 13.5 g/dL in men and <11.0 g.dL in women), small bowel obstruction, new-onset perianal disease, or intra-abdominal abscess. Secondary outcomes were measured between 6 and 12 months after the initial zinc level measurement. Outcomes were assessed in the entire cohort and in the UC and CD subgroups individually.

### 2.3. Statistical Analysis

Descriptive statistics including numbers and percentages were used for categorical data. Continuous variables were summarized using mean with standard deviation (SD) or median with interquartile ranges (IQRs). Normality of quantitative variables was tested using the Shapiro–Wilk test. The Chi-Square test and Wilcoxon rank sum test were used to examine the differences in characteristics between groups. Univariable logistic regression was conducted to explore the predictors of secondary outcomes including the rate IBD-related hospitalizations, ER visits, IBD-related surgeries, and IBD-related complications. Multivariate analysis was performed to assess the relationship between multiple predictors to the secondary outcomes. In the multivariate model, we included factors that demonstrated a *p*-value ≤ 0.1 in the univariate analysis. Results of both univariable and multivariable logistic regression were reported as odds ratios (ORs) with 95% confidence intervals (CIs). Complete-case sensitivity analysis was utilized for missing data. Data analysis was performed using Stata version 18 (StataCorp, College Station, TX, USA). All tests were two-tailed, and statistical significance was set at a *p*-value < 0.05.

## 3. Results

### 3.1. Study Population

**Figure 1:**
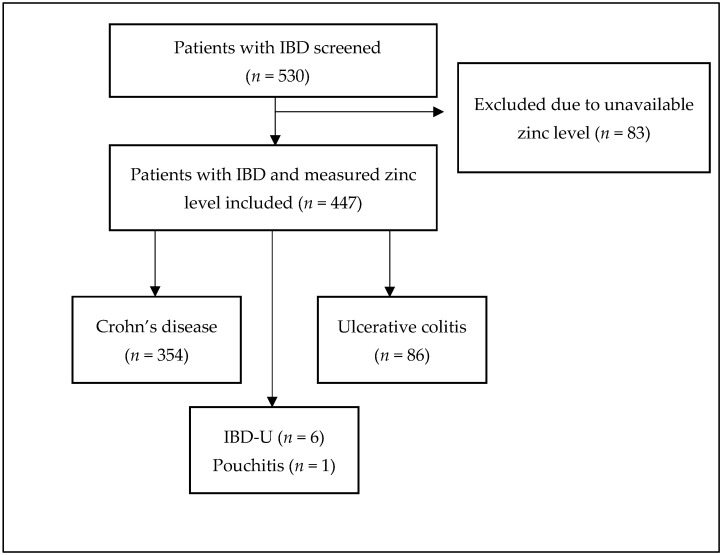
Flow diagram illustrating patients screened, excluded and included in the study.

A total of 530 patients with IBD were initially screened, of whom 447 were included after excluding those lacking documented zinc level measurements. Most of the patients had CD at 79.2% (*n* = 354), followed by UC at 19.3% (*n* = 86), IBD-U 1.3% (*n* = 6), and pouchitis 0.2% (*n* = 1) (Figure 1). The median age of the cohort was 29 years (IQR: 22–38); median disease duration was 5.5 years (IQR: 2–12); and 54.4% (*n* = 243) were male. The most common disease location in CD was ileocolonic (73.7%) and most common phenotype was penetrating disease (43.5%), with 38.3% having perianal disease. The most common UC location was pancolitis (63.9%). Extraintestinal manifestations were noted in 11.6% (*n* = 52) of the entire cohort. History of intestinal resection was noted in 28.6% (*n* = 128) of the cohort (Table 1).

A total of 211 patients (47.2%) were on immunomodulator therapies which included thiopurines (*n* = 207) and methotrexate (*n* = 8). Advanced therapies were noted in 237 patients (53.0%) including adalimumab (*n* = 138), infliximab (*n* = 57), ustekinumab (*n* = 23), vedolizumab (*n* = 16), tofacitinib (*n* = 5), and certolizumab (*n* = 2). Additionally, 72 patients (16.1%) were on corticosteroids at the time of zinc level measurement.

At baseline (time of zinc level measurement), 36% (*n* = 161) were in clinical remission as per documented treating physician global assessment. Baseline endoscopic data (within 30 days of zinc level measurement) was available for 229 patients, in which 31% were in endoscopic remission (defined as absence of erosions/ulcerations in CD and Mayo UC endoscopic sub-score ≤ 1 in UC).

### 3.2. Primary Outcomes

**Figure 2:**
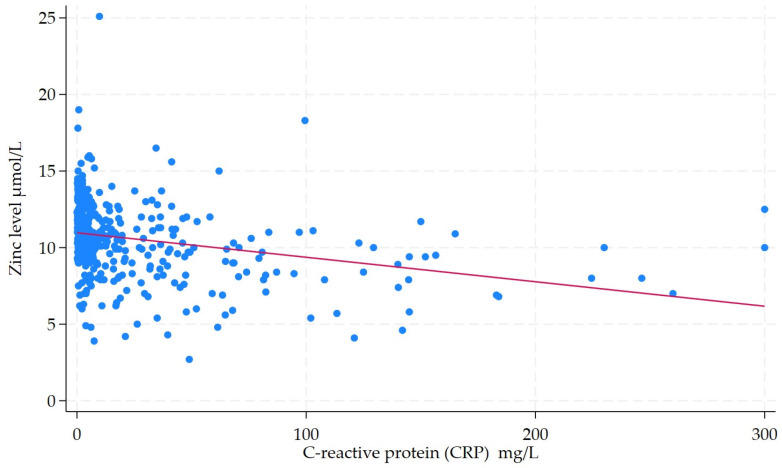
Scatter plot with fitted regression line illustrating the relationship between C-reactive protein (CRP) and zinc levels in patients with IBD. Each blue dot represents individual patient’s data point, showing that patient’s measured CRP level and corresponding zinc level. The red line is the line of best fit (regression line), which summarizes the overall trend or correlation between CRP and zinc levels.

**Figure 3:**
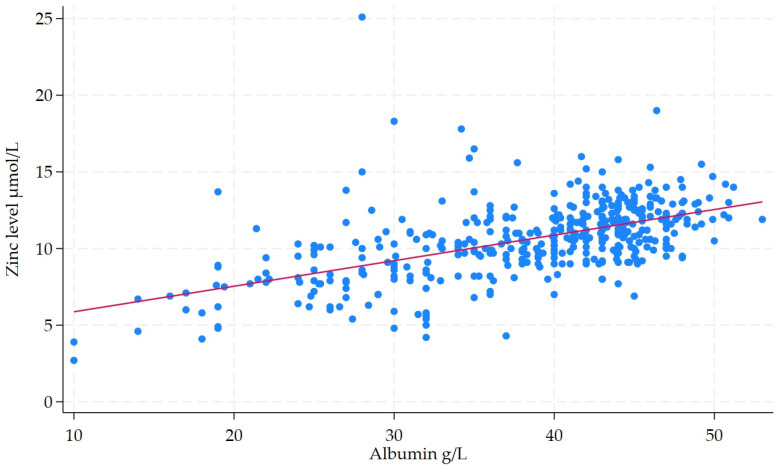
Scatter plot with fitted regression line illustrating the relationship between albumin and zinc levels in patients with IBD. Each blue dot represents individual patient’s data point, showing that patient’s measured CRP level and corresponding zinc level. The red line is the line of best fit (regression line), which summarizes the overall trend or correlation between CRP and zinc levels.

The median baseline zinc level for the entire cohort was 10.7 (IQR: 9.3–12.1). Zinc deficiency was noted in 45.6% (*n* = 204) of the entire cohort. The rate of zinc deficiency was 48.3% in CD and 36.0% in UC. Zinc levels demonstrated a weak negative correlation with CRP levels (*r*^2^ = 0.08, *p* < 0.001) (Figure 2). However, zinc levels did show a moderate positive relationship with albumin levels (*r*^2^ = 0.31, *p* < 0.001) (Figure 3). The rate of zinc deficiency was lower in patients in clinical remission at 29.8% (48/161) vs. 63.5% (115/181) in those with clinically active IBD (*p* < 0.001). In addition, zinc deficiency was also less common in patients in endoscopic remission at 45.1% (32/71) vs. 60.1% (95/158) in those with endoscopically active diseases (*p* = 0.034).

Patients with zinc deficiency were more likely to be female (*p* = 0.04), be of younger age (*p* < 0.001), have shorter disease duration (*p* < 0.001), and less likely to be smokers (*p* = 0.002) (Table 2). The median CRP was significantly higher in the zinc deficiency group (12.1 vs. 3.4, *p* < 0.001). Median FCP levels were also significantly higher in the zinc deficiency group (660 vs. 177, *p* < 0.001) while the median albumin levels were significantly lower in the zinc deficiency group (35.2 vs. 43.3, *p* < 0.001). Patients with zinc deficiency were more likely to be on corticosteroids compared to patients without zinc deficiency (20.1% vs. 12.8%, *p* = 0.04).

### 3.3. Secondary Outcomes

**Figure 4:**
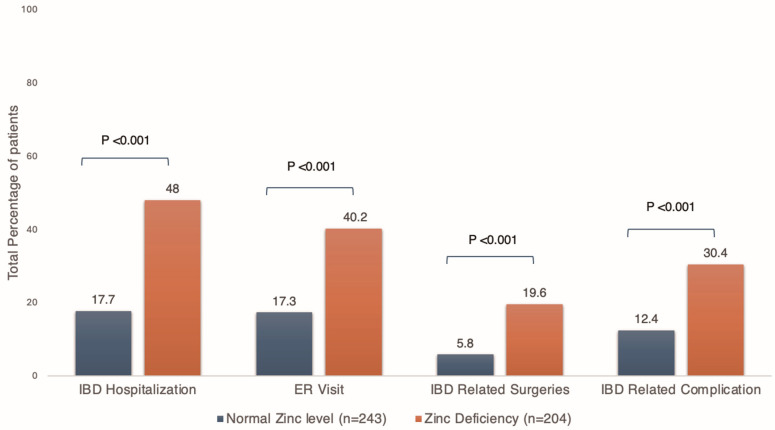
Bar graph depicting rates of secondary outcomes in patients with and without zinc deficiencies.

IBD-related hospitalization occurred in 31.5% (*n* = 141) of the entire cohort. ER visits were noted in 27.8% of patients (*n* = 124), IBD-related surgeries in 12.1% (*n* = 54), and IBD-related complications in 20.6% (*n* = 92). IBD-related hospitalization rates were 48% vs. 17.7% (*p* < 0.001) in patients with and without zinc deficiency, respectively. Patients with zinc deficiencies had higher rates of ER visits (40.2% vs. 17.3%, *p* < 0.001), IBD-related surgeries (19.6% vs. 5.8%, *p* < 0.001), and IBD-related complications (30.4% vs. 12.4%, *p* < 0.001) (Figure 4). The breakdown of specific IBD-related complications in the cohort is shown in (Table 3).

Univariate analyses were performed to identify factors and variables associated with secondary outcomes including IBD-related hospitalizations, ER visits, IBD-related surgeries and IBD-related complications. On univariate analysis, young age (*p* < 0.005), shorter disease duration (*p* < 0.005), CD subtype (*p* = 0.041), zinc deficiency (*p* < 0.005), high CRP (*p* < 0.005), high FCP (*p* < 0.005), low albumin (*p* < 0.005) and corticosteroids (*p* < 0.005) were significantly associated with IBD-related hospitalizations but multivariable analysis showed that only zinc deficiency (OR 2.42, 95% CI 1.07–5.48, *p* = 0.033) and low albumin (OR 9.03. 95% CI 3.38–24.15, *p* < 0.001) were independently associated with IBD-related hospitalizations (Supplementary Table S1). In terms of IBD-related ER visits, young age (*p* = 0.006), shorter disease duration (*p* < 0.005), zinc deficiency (*p* < 0.001), high CRP (*p* < 0.001), high FCP (*p* < 0.001), low albumin (*p* < 0.001), and corticosteroids (*p* = 0.002) were predictive risk factors, but only low albumin (OR 4.56, 95% CI 1.81–11.52, *p* = 0.001) was independently associated with IBD-related ER visits of multivariable analysis (Supplementary Table S2).

Upon univariate analysis, CD subtype (*p* < 0.001), zinc deficiency (*p* < 0.001), high CRP (*p* < 0.001), high FCP (*p* = 0.031), low albumin (*p* < 0.001), and corticosteroids (*p* = 0.013) were predictive of IBD-related surgeries but multivariate analysis showed that only zinc deficiency (OR 5.23, 95% CI 1.27–21.45, *p* = 0.022) and CD subtype (OR 9.38, 95% CI 1.12–78.85, *p* = 0.039) were independently associated with IBD-related surgeries (Supplementary Table S3). IBD-related complications were more common in patients with CD subtype (*p* = 0.041), perianal CD (*p* = 0.011), zinc deficiency (*p* < 0.001), high CRP (*p* < 0.001), high FCP (*p* = 0.026), and low albumin (*p* < 0.001). Multivariate analysis showed that only zinc deficiency (OR 3.98. 95% CI 1.53–10.41, *p* = 0.005) and CD subtype (OR 3.3, 95% CI 1.01–10.84, *p* = 0.049) were predictive of IBD-related complications (Supplementary Table S4).

### 3.4. Subgroup Analysis

Univariate and multivariate analyses were performed for subgroups of Crohn’s disease (Supplementary Tables S5–S8) and ulcerative colitis (Supplementary Tables S9–S11) patients. In the CD cohort, only low albumin was associated with hospitalizations (*p* < 0.001) and ER visits (*p* = 0.003), while zinc deficiency was independently associated with surgeries in CD (*p* = 0.022) and IBD-related complications (*p* = 0.014), as indicated from the multivariate analysis. In the UC cohort, zinc deficiency was not associated with hospitalizations, ER visits or surgeries.

## 4. Discussion

In this study, we evaluated the prevalence, clinical characteristics and outcomes of zinc deficiency in a well-characterized cohort of patients with IBD. Overall, the rate of zinc deficiency was relatively high at 45.6% with a noted higher prevalence in the CD cohort compared to UC. Zinc levels did correlate with markers of inflammation including CRP and albumin. Zinc deficiency was associated with unfavorable clinical outcomes including IBD-related hospitalizations, IBD-related surgeries, and IBD-related complications, even after controlling for markers of inflammation. Zinc deficiency’s impact on clinical outcomes was more pronounced mainly in the CD cohort compared to the UC cohort, likely due to the fact that inflammation in CD often involves the small intestine, which is a primary site for the absorption of key micronutrients [19].

Zinc has multiple physiological roles including but not limited to the maintenance of the intestinal barrier function, wound healing, cellular metabolism, and protein synthesis [20]. The prevalence of zinc deficiency reported in the literature in IBD has been variable. A recent systematic review and metanalysis of 17 studies (*n* = 2413), including 9 studies on CD (*n* = 1677) and 8 studies on UC (*n* = 736), reported on the prevalence of zinc deficiency in IBD patients [10]. The overall prevalence of zinc deficiency was 50% (95% CI 0.48–0.52), similar to the prevalence observed in our cohort using comparable cut-off levels. As in our cohort, the prevalence of zinc deficiency was higher in CD patients at 54% (95% CI 0.51–0.56) compared to 41% (95% CI 0.38–0.45) in UC patients [10]. This highlights the relatively high prevalence of zinc deficiency in IBD patients and the importance of investigating zinc levels in patients with IBD in clinical practice. The European Society for Clinical Nutrition and Metabolism (ESPEN) devised updated guidelines on clinical nutrition in IBD in 2023 [21].

The guidelines recommend checking micronutrient levels such as zinc regularly in all patients with IBD, including those who are in remission [21]. This recommendation is consistent with the findings of our study, as zinc deficiency was noted up to 30% of patients in clinical remission and 45% of those in endoscopic remission.

Serum zinc levels are lower in the setting of active inflammation, which points to its inherent property as a negative acute phase reactant [22,23]. However, the strength of correlation between disease activity and zinc levels is variable. In our cohort, we have demonstrated a weak correlation between zinc levels and CRP and a moderate correlation between zinc and albumin levels. A retrospective study of 232 patients with IBD (155 with CD and 77 with UC) failed to show a correlation between zinc levels and disease activity as measured by clinical and deep remission (a composite of clinical, endoscopic, and biochemical remission with CRP and FCP) [15]. Therefore, zinc deficiency in IBD is likely multifactorial and not only related to being a negative acute phase reactant during active inflammation as noted by the relatively high prevalence of zinc deficiency in IBD patients in endoscopic remission in our cohort. Zinc deficiency in IBD may occur due to enteric losses in the setting of chronic diarrhea, previous bowel surgeries, high ostomy output, decreased oral intake, and malabsorption [23].

Several factors are associated with malnutrition in IBD patients. Reduced oral intake and poor appetite often result from IBD-related gastrointestinal symptoms. Chronic diarrhea and ongoing inflammation can lead to both protein and blood loss, while structural changes in the gut, such as impaired epithelial transport and compromised mucosal integrity, further impair nutrient absorption. Surgical interventions, particularly ileal resection, also play a major role in malabsorption. Additionally, small intestinal bacterial overgrowth (SIBO), which can develop in IBD patients, can markedly affect nutrient absorption [7]. A couple of isotopic zinc absorption studies have demonstrated impairment of zinc absorption in patients with CD [11,12].

Our study demonstrated an association between baseline zinc deficiency and unfavorable clinical outcomes including risk of IBD-related hospitalizations, IBD-related surgeries, and IBD-related complications. Siva et al. investigated the clinical outcomes of 996 patients with IBD (773 with CD and 223 with UC) in relation to serum zinc level status [14]. As in our study, zinc deficiency was associated with the female sex, high CRP values, and low albumin levels. In terms of outcomes, the study by Siva et al. demonstrated an association between zinc deficiency and IBD-related hospitalizations, surgeries, and complications. The authors of this study also controlled for markers of inflammation, including CRP and albumin, as we did in our study, emphasizing the independent impact of zinc deficiency on unfavorable clinical outcomes, particularly in CD. In a separate retrospective cohort of 216 patients with IBD, zinc deficiency was also associated with a higher risk of surgery (*p* = 0.002) and treatment with corticosteroids (*p* < 0.001) in patients with CD [19]. These findings are in congruence with the findings of our study emphasizing the association of zinc deficiency with poor clinical outcomes. It is unclear whether oral zinc supplementation results in improved outcomes in patients with IBD and zinc deficiency. In a small retrospective cohort of 51 patients with IBD and zinc deficiency, oral zinc replacement resulted in an improvement in zinc levels and a significant improvement in clinical disease activity in CD, as measured by the Crohn’s disease activity index (CDAI) [24]. Oral zinc replacement in patients with UC, however, was not associated with changes in clinical disease activity [24].

There is minimal data on the underlying mechanism of the negative impact of zinc deficiency on outcomes in IBD. Epidemiologic studies have suggested an inverse relationship between dietary zinc intake and CD incidence. In an analysis of 170,776 women from the Nurses Health Study I and Nurses Health Study II, high dietary and supplemental zinc intake was associated with a lower risk of CD but not UC [25]. There have been multiple IBD animal-based experimental models that aimed to investigate the therapeutic mechanisms of zinc supplementation on colitis. Proposed mechanisms in which zinc may potentially exert its therapeutic impact include modulation of the intestinal barrier, immune system function, acting as antioxidant, and positively impacting the intestinal microbiome [26]. Li et al. demonstrated that zinc nanoparticle treatment in a dose-dependent fashion induced remission in dextran sulfate sodium-induced ulcerative colitis in mice through direct anti-inflammatory properties [27]. In addition, zinc nanoparticle treatment activated Nuclear Factor Erythroid 2-Related Factor (Nrf2) in the cellular antioxidant pathway [27].

Our study results highlight the importance of nutritional assessment in IBD patients and support the implementation of periodic monitoring of serum zinc levels, particularly for patients with active Crohn’s disease. Our study has multiple strengths. First, we describe a very well-characterized and relatively large cohort of patients with IBD and zinc deficiency. Due to the granular data available on our cohort, we were able to attempt to control for markers inflammation including CRP and albumin while analyzing the impact of zinc deficiency on clinical outcomes. We were also able to conduct subgroup analysis highlighting that the role of zinc deficiency is more pronounced in CD vs. UC. Our study also has multiple limitations. This includes inherent limitations of the retrospective single-center study design, including selection bias and missing data. Although we have attempted to control for markers of inflammation via multivariable analysis, there could be confounding bias of unmeasured variables of disease activity and severity. In addition, we relied on serum zinc levels to define deficiency; serum zinc levels can exhibit circadian variability and is dependent on multiple factors including diet and hormones [28]. In addition, due to the limited number of patients with purely colonic Crohn’s disease, the association between zinc deficiency and disease location could not be fully explored. Finally, based on our data, we were not able to evaluate the impact of oral zinc supplementation and normalization of zinc serum levels on clinical outcomes.

In conclusion, zinc deficiency is highly prevalent in IBD patients and particularly prevalent in CD patients. Low serum zinc levels have weak to moderate correlations with serum markers of inflammation. Zinc deficiency is associated with selected poor clinical outcomes that are independent of serum markers of inflammation. The impact of zinc deficiency on clinical outcomes was pronounced in CD rather than in UC. Future prospective and interventional studies assessing the impact of oral zinc supplementation and the correction of zinc deficiency on clinical outcomes in IBD are warranted to establish causality and clarify zinc’s role in disease progression.

## Figures and Tables

**Table 1:** Baseline characteristics of the entire cohort included in the analysis.

Variable	Value
Age (years), median (IQR)	29 (22–38)
Male, *n* (%)	243 (54.4)
Disease duration (years), median (IQR)	5.5 (2–12)
Follow-up time (months), median (IQR)	
IBD subtype	
Crohn’s disease, *n* (%) *	354 (79.2)
L1	56 (15.8)
L2	34 (9.6)
L3	261 (73.7)
L4	8 (2.3)
B1	105 (29.7)
B2	64 (18.2)
B3	154 (43.5)
B2 + B3	30 (8.5)
Perianal disease, *n* (%)	136 (38.3)
Ulcerative colitis, *n* (%)	86 (19.3)
Proctitis	9 (10.5)
Left-sided colitis	22 (25.6)
Pancolitis	55 (63.9)
Pouchitis	1 (0.2)
IBD-U	6 (1.3)
Prior bowel resection, *n* (%)	128 (28.6)
Current smoker, *n* (%)	48/297 (16.2)
Extraintestinal manifestation, *n* (%)	52 (11.6)

* 1 patient with isolated perianal disease. IBD: Inflammatory bowel disease; IBD-U: inflammatory bowel disease-unclassified; IQR: interquartile range; BMI: body mass index.

**Table 2:** Baseline characteristics of IBD patients with normal and deficient zinc levels.

Characteristics	Normal Zinc Level (*n* = 243)	Zinc Deficiency (*n* = 204)	OR (95% CI)	*p*-Value
Male, *n* (%)	143 (58.9)	100 (49.0)	1.48 (1.02–2.16)	**0.04**
Age, median (IQR)	30 (24–41)	27 (21–35.8)	0.97 (0.95–0.98)	**<0.001**
Disease duration, median (IQR)	7 (3–13)	4 (1–9)	0.94 (0.91–0.97)	**<0.001**
Smoking, *n* (%) *	35 (22.3)	13 (9.3)	0.36 (0.18–0.71)	**0.002**
Previous surgery, *n* (%)	72 (29.6)	56 (27.5)	0.89 (0.59–1.36)	0.61
Disease subtype, *n* (%)				0.14
Crohn’s disease	183 (75.3)	171 (83.8)		
Ulcerative colitis	55 (22.6)	31 (15.2)	0.60 (0.37–0.98)	
IBD-U	4 (1.7)	2 (1.0)	0.53 (0.097–2.96)	
Pouchitis	1 (0.4)	0		
Perianal Crohn’s disease, *n* (%)	69 (28.4)	67 (32.8)	1.23 (0.82–1.85)	0.31
Extraintestinal Manifestations, *n* (%)	25 (10.3)	27 (13.2)	1.33 (0.74–2.37)	0.33
**Labs**				
Median CRP (IQR)	3.4 (0.9–10.6)	12.1 (3.8–46.8)	3.34 (2.24–4.99)	**<0.001**
Median FCP (IQR)	177 (49–750)	660 (146–1000)	2.79 (1.57–4.96)	**<0.001**
Median Albumin (IQR)	43.3 (40.4–45.7)	35.2 (28–40.5)	7.84 (4.73–12.99)	**<0.001**
**Medications**				
Immunomodulators, *n* (%)	113 (46.5)	98 (48.0)	1.06 (0.73–1.54)	0.75
Advanced Therapies, *n* (%)	128 (52.7)	109 (53.4)	1.03 (0.71–1.49)	0.87
Corticosteroids, *n* (%)	31 (12.8)	41 (20.1)	1.72 (1.03–2.86)	**0.04**

* Smoking status was known in 297 of 447 patients. IQR: interquartile range; IBD-U: inflammatory bowel disease-unclassified; CRP: C-reactive protein; FCP: fecal calprotectin, OR: odds ratio; CI: confidence interval. Bold indicates *p* < 0.05.

**Table 3:** Association of zinc level status with IBD-related complications.

Outcome	Normal Zinc Level (*n* = 243)	Zinc Deficiency (*n* = 204)	*p*-Value
IBD-related complications, *n* (%)	30 (12.4)	62 (30.4)	**<0.001**
New-onset perianal fistula, *n* (%)	2 (0.8)	18 (8.8)	**<0.001**
Small bowel obstruction, *n* (%)	3 (1.2)	17 (8.3)	**<0.001**
New-onset anemia, *n* (%)	18 (7.4)	26 (12.8)	0.06
Intra-abdominal abscess, *n* (%)	7 (2.88)	18 (8.8)	**0.006**

IBD: inflammatory bowel disease. Bold indicates *p* < 0.05.

## Data Availability

The data that support the findings of this study are available from the corresponding author upon reasonable request.

## Supplementary Materials

The following supporting information can be downloaded at https://www.mdpi.com/article/10.3390/nu17213378/s1. Supplementary Table S1: Univariate and Multivariable Analysis on Predictors of IBD-Related Hospitalizations. Supplementary Table S2: Univariate and Multivariable Analysis on Predictors of IBD-Related Emergency Room Visits. Supplementary Table S3: Univariate and Multivariable Analysis on Predictors of IBD-Related Surgery. Supplementary Table S4: Univariate and Multivariable Analysis on Predictors of IBD-Related Complications. Supplementary Table S5: Univariate and Multivariate Analyses on Predictors of IBD-related Hospitalization in Crohn’s Disease. Supplementary Table S6: Univariate and Multivariate Analyses on Predictors of IBD-related Emergency Room Visits in Crohn’s Disease. Supplementary Table S7: Univariate and Multivariate Analyses on Predictors of IBD-related Surgeries in Crohn’s Disease. Supplementary Table S8: Univariate and Multivariate Analyses on Predictors of IBD-related Complications in Crohn’s Disease. Supplementary Table S9: Univariate and Multivariate Analyses on Predictors of IBD-related Hospitalizations in Ulcerative Colitis. Supplementary Table S10: Univariate and Multivariate Analyses on Predictors of IBD-related Emergency Room Visits in Ulcerative Colitis. Supplementary Table S11: Predictors of IBD-related Surgeries in Ulcerative Colitis.
