# Supplementary material for: Prevalence and Impact of Zinc Deficiency on Clinical Outcomes in Inflammatory Bowel Disease
Source: Nutrients. 2025 Oct 28;17(21):3378. doi: 10.3390/nu17213378 (PMC12610508; doi:10.3390/nu17213378)
Supplement: Supplementary file 1 [file nutrients-17-03378-s001.zip › nutrients-3926373-supplementary.pdf]

SUPPLEMENTARTY TABLES

Table S1: Univariate and Multivariable Analysis on Predictors of IBD-Related Hospitalizations

Characteristics	Univariable analysis				Multivariable analysis	
	Hospitalization		OR (95% CI)	p-value	OR (95% CI)	p-value
	No (n=306)	Yes (n=141)				
**Male, n (%)**	168 (54.9)	75 (53.19)	1.07 (0.71-1.60)	0.736		
**Age, years, median (IQR)**	30 (23-40)	27 (21-34)	0.97 (0.95-0.99)	**<0.005**	0.99 (0.96-1.04)	0.872
**Disease duration, years, median (IQR)**	6 (3-12.5)	4 (1-7)	0.94 (0.90-0.97)	**<0.005**	0.99 (0.92-1.06)	0.768
**Smoking, n (%)***	33 (17.0)	15 (14.6)	0.83 (0.43-1.61)	0.586		
**Previous surgery, n (%)**	88 (28.76)	40 (28.37)	0.98 (0.63-1.53)	0.933		
**Disease subtype, n (%)**	306	141		**0.041**		
**Crohn's disease**	231(75.5)	123 (87.2)				
**Ulcerative colitis**	69 (22.6)	17 (12.1)	0.46 (0.26-0.82)		0.60 (0.23-1.59)	0.306
**IBD-U**	5 (1.6)	1 (0.7)	0.37 (0.043-3.25)		1.83 (0.17-20.14)	0.62
**Pouchitis**	1 (0.3)	0				
**Perianal Crohn's disease, n (%)**	85 (27.8)	51 (36.2)	1.47 (0.96-2.25)	0.073	1.09 (0.43-2.75)	0.863
**Extraintestinal Manifestations, n (%)**	32 (10.5)	20 (14.2)	1.42 (0.78-2.57)	0.253		
**Labs**						
**Zinc deficiency, n (%)**	106 (34.6)	98 (69.5)	4.30 (2.80-6.60)	**<0.005**	2.42 (1.07-5.48)	**0.033**
**High CRP, n (%)**	107 (37.3)	114 (83.2)	8.34 (5.02-13.86)	**<0.005**	2.07 (0.86-4.98)	0.105
**High FCP, n (%)**	76 (48.7)	46 (48.7)	4.40 (2.12-9.12)	**<0.005**	1.21 (0.48-3.06)	0.678
**Low Albumin, n (%)**	36 (12.3)	83 (59.3)	10.35 (6.37-16.82)	**<0.005**	9.03 (3.38-24.15)	**<0.001**
**Medications**						
**Immunomodulators, n (%)**	146 (47.7)	65 (46.1)	0.94 (0.63-1.39)	0.751		
**Advanced Therapies, n (%)**	159 (51.9)	78 (55.3)	1.14 (0.77-1.71)	0.509		
**Corticosteroids, n (%)**	36 (11.8)	36 (25.5)	2.57 (1.54-4.29)	**<0.005**	1.32 (0.49-3.51)	0.584

IQR: interquartile range; IBD-U: inflammatory bowel disease-unclassified; CRP: C-reactive protein; FCP: fecal calprotectin; OR: odds ratio; CI: confidence interval
**Smoking status was known in 297 of 447 patients*

Table S2: Univariate and Multivariable Analysis on Predictors of IBD-Related Emergency Room Visits

**Characteristics**	**Univariable analysis**				**Multivariable analysis**	
	**Emergency Room Visits**		**OR (95% CI)**	**p-value**	**OR (95% CI)**	**p-value**
	**No (n=323)**	**Yes (n=124)**				
**Male, n (%)**	174 (53.9)	68 (55.7)	0.93 (0.61-1.41)	0.736		
**Age, years, median (IQR)**	30 (23-39)	26 (21-34.5)	0.97 (0.95-0.99)	**0.006**	0.99 (0.96-1.03)	0.750
**Disease duration, years, median (IQR)**	6 (2-12)	4 (1-7)	0.94 (0.90-0.97)	**<0.005**	0.97 (0.9-1.05)	0.436
**Smoking, n (%) ***	38 (18.1)	10 (11.5)	0.59 (0.28-1.24)	0.160		
**Previous surgery, n (%)**	90 (27.9)	38 (30.7)	1.14 (0.73-0.79)	0.560		
**Disease subtype, n (%)**				0.143		
**Crohn's disease**	247 (76.5)	107 (86.3)	Reference	**-**		
**Ulcerative colitis**	70 (21.7)	16 (12.9)	0.53 (0.29-0.95)	**0.033**		
**IBD-U**	5 (1.6)	1 (0.3)	0.46 (0.05-3.99)	0.483		
**Pouchitis**	1(0.8)	0	-	**-**		
**Perianal Crohn's disease, n (%)**	90 (27.9)	38 (30.7)	1.25 (0.80-1.94)	0.560		
**Extraintestinal Manifestations, n (%)**	32 (9.9)	20 (16.1)	1.75 (0.96-3.19)	0.066	1.85 (0.61-5.55)	0.275
**Labs**						
**Zinc deficiency, n (%)**	122 (37.8)	82 (66.1)	3.22 (2.08-4.97)	**<0.001**	1.67 (0.72-3.87)	0.234
**High CRP, n (%)**	123 (40.5)	98 (81.7)	6.56 (3.91-10.98)	**<0.001**	2.10 (0.85-5.18)	0.108
**High FCP, n (%)**	83 (50.0)	39 (82.9)	4.88 (2.15-11.06)	**<0.001**	1.67 (0.62-4.5)	0.307
**Low Albumin, n (%)**	47 (15.2)	72 (58.5)	7.87 (4.89-12.65)	**<0.001**	4.56 (1.81-1.52)	**0.001**
**Medications**						
**Immunomodulators, n (%)**	156 (48.3)	55 (44.4)	0.85 (0.56-1.29)	0.455	1.16 (0.44-3.05)	0.77
**Advanced Therapies, n (%)**	167 (51.7)	70 (56.5)	1.21 (0.79-1.84)	0.368		
**Corticosteroids, n (%)**	41(12.7)	31(25.0)	2.29 (1.36-3.86)	**0.002**	1.16 (0.44-3.05)	0.77

IQR: interquartile range; IBD-U: inflammatory bowel disease-unclassified; CRP: C-reactive protein; FCP: fecal calprotectin; OR: odds ratio; CI: confidence interval
**Smoking status was known in 297 of 447 patients*

<u>Table S3:</u> Univariate and Multivariable Analysis on Predictors of IBD-Related Surgery

**Characteristics**	**Univariable analysis**				**Multivariable analysis**	
	**IBD-Related Surgery**		**OR (95% CI)**	**p-value**	**OR (95% CI)**	**p-value**
	**No (n=393)**	**Yes (n=54)**				
**Male, n (%)**	218 (55.5)	25 (46.3)	0.93 (0.61-1.41)	0.204		
**Age, years, median (IQR)**	29 (23-38)	27.5 (20-34)	0.97 (0.95- 0.99)	0.227		
**Disease duration, years, median (IQR)**	6 (2 -12)	4 (1-7)	0.94 (0.90-0.97)	0.085	0.99(0.91-1.08)	0.875
**Smoking, n (%) ***	43 (16.7)	5 (12.5)	0.59 (0.28- 1.24)	0.499		
**Previous surgery, n (%)**	112 (28.5)	16 (29.6)	1.14 (0.73-1.79)	0.863		
**Disease subtype, n (%)**				**<0.001**		
**Crohn's disease**	301(76.6)	53 (98.2)			9.38(1.12-78.85)	**0.039**
**Ulcerative colitis**	85 (21.6)	1(1.9)	0.53 (0.29-0.95)	**0.033**		
**IBD-U**	6 (1.53)	0	0.46 (0.05-3.99)	0.483		
**Pouchitis**	1 (0.25)	0				
**Perianal Crohn's disease, n (%)**	114 (29.0)	22 (40.7)	1.25 (0.80-1.94)	0.327	1.22 (0.42-3.5)	0.718
**Extraintestinal Manifestations, n (%)**	46 (11.7)	6 (11.1)	1.75 (0.96-3.19)	0.898		
**Labs**						
**Zinc deficiency, n (%)**	164 (41.7)	40 (74.1)	3.2 (2.08-4.97)	**<0.001**	5.23 (1.27-21.45)	**0.022**
**High CRP, n (%)**	173 (46.6)	48 (90.6)	6.55 (3.91-10.98)	**<0.001**	2.63 (0.63-10.95)	0.185
**High FCP, n (%)**	104 (54.7)	18 (78.3)	4.87 (2.14-11.06)	**0.031**	0.68 (0.16-2.97)	0.611
**Low Albumin, n (%)**	90 (23.8)	29 (53.7)	7.87 (4.89-12.65)	**<0.001**	3.42 (0.99-11.78)	0.051
**Medications**						
**Immunomodulators, n (%)**	181 (46.1)	30 (55.6)	0.85 (0.56-1.29)	0.190		
**Advanced Therapies, n (%)**	204 (51.9)	33 (61.1)	1.21 (0.79-1.84)	0.204		
**Corticosteroids, n (%)**	57 (14.5)	15 (27.8)	2.29 (1.36-3.86)	**0.013**	0.89 (0.26-3.03)	0.854

IQR: interquartile range; IBD-U: inflammatory bowel disease-unclassified; CRP: C-reactive protein; FCP: fecal calprotectin; OR: odds ratio; CI: confidence interval
**Smoking status was known in 297 of 447 patients*

Table S4: Univariate and Multivariable Analysis on Predictors of IBD-Related Complications

Characteristics	Univariable analysis				Multivariable analysis	
	IBD-Related Complications		OR (95% CI)	p-value	OR (95% CI)	p-value
	No (n=355)	Yes (n=92)				
**Male, n (%)**	192 (54.1)	51 (55.4)	0.94 (0.59-1.50)	0.817		
**Age, years, median (IQR)**	29 (23- 38)	27 (21- 39)	0.99 (0.97-1.01)	0.404		
**Disease duration, years, median (IQR)**	6 (2-12)	5 (1-12)	0.99 (0.96-1.02)	0.463		
**Smoking, n (%) ***	40 (18.0)	8 (10.7)	0.54 (0.24-1.22)	0.135		
**Previous surgery, n (%)**	100 (28.2)	28 (30.4)	1.11 (0.68-1.84)	0.668		
**Disease subtype, n (%)**				**0.041**	3.3 (1.01-10.84)	**0.049**
**Crohn's disease**	272 (76.6)	82 (89.1)				
**Ulcerative colitis**	76 (21.4)	10 (10.9)	0.44 (0.22-0.88)			
**IBD-U**	6 (1.7)	0				
**Pouchitis**	1 (0.3)	0				
**Perianal Crohn's disease, n (%)**	98 (27.6)	38 (41.3)	1.84 (1.15-2.969)	**0.011**	1.41 (0.58-3.39)	0.446
**Extraintestinal Manifestations, n (%)**	39 (10.9)	13 (14.1)	1.33 (0.68- 2.617)	0.402		
**Labs**						
**Zinc deficiency, n (%)**	142 (40.0)	62 (67.4)	3.1 (1.91-5.03)	**<0.001**	3.98 (1.53-10.41)	**0.005**
**High CRP, n (%)**	159 (47.3)	62 (70.5)	2.65 (0.60-4.40)	**<0.001**	0.91 (0.33-2.47)	0.852
**High FCP, n (%)**	96 (53.9)	26 (74.3)	2.47 (1.09-5.56)	**0.026**	1.75 (0.62-4.94)	0.290
**Low Albumin, n (%)**	76 (22.2)	43 (47.8)	3.20 (1.97-5.20)	**<0.001**	1.74 (0.64-4.76)	0.277
**Medications**						
**Immunomodulators, n (%)**	164 (46.2)	47 (51.1)	1.22 (0.77-1.92)	0.402		
**Advanced Therapies, n (%)**	193 (54.4)	44 (47.8)	0.77 (0.49 -1.217)	0.263		
**Corticosteroids, n (%)**	55 (15.5)	17 (18.5)	1.24 (0.68-2.25)	0.488		

IQR: interquartile range; IBD-U: inflammatory bowel disease-unclassified; CRP: C-reactive protein; FCP: fecal calprotectin; OR: odds ratio; CI: confidence interval
**Smoking status was known in 297 of 447 patients*

Table S5: Univariate and Multivariate Analyses on Predictors of IBD-related Hospitalization in Crohn's Disease.

**Variable**	**Univariate analysis**		**Multivariate analysis**	
	**Odds ratio (95% CI)**	**P-value**	**Odds ratio (95% CI)**	**P-value**
**Male**	1.35 (0.87-2.10)	0.185		
**Age**	0.98 (0.96-1)	0.02	1 (0.96-1.05)	0.95
**Disease duration**	0.94 (0.9-0.98)	<0.001	0.99 (0.91-1.07)	0.75
**Smoking**	0.72 (0.36-1.45)	0.36		
**Previous surgery**	0.74 (0.46-1.18)	0.21		
**Perianal Crohn's disease**	1.24 (0.79-1.94)	0.35		
**Extraintestinal Manifestations**	1.03 (0.49-2.15)	0.94		
**Labs**				
**Zinc deficiency**	3.99 (2.5-6.38)	<0.001	1.76 (0.64-4.82)	0.28
**High CRP**	8.74 (4.94-15.46)	<0.001	2.65 (0.92-7.64)	0.07
**High FCP**	4.42 (1.92-10.15)	<0.001	0.97 (0.33-2.82)	0.95
**Low Albumin**	9.27 (5.45-15.75)	<0.001	12.76 (3.67-44.39)	**<0.001**
**Medications**				
**Immunomodulators**	0.96 (0.62-1.49)	0.87		
**Advanced Therapies**	0.84 (0.54-1.3)	0.43		
**Corticosteroids**	2.34 (1.32-4.14)	<0.001	1.15 (0.35-3.75)	0.82

CRP: C-reactive protein; FCP: fecal calprotectin

Table S6: Univariate and Multivariate Analyses on Predictors of IBD-related Emergency Room Visits in Crohn's Disease.

Variable	Univariate analysis		Multivariate analysis	
	**Odds ratio (95% CI)**	**P-value**	**Odds ratio (95% CI)**	**P-value**
**Male**	1.16 (0.73 - 1.83)	0.533		
**Age**	0.98 (0.96 – 1)	0.027	0.99 (0.95 - 1.04)	0.819
**Disease duration**	0.94 (0.9 - 0.98)	0.002	0.96 (0.89 - 1.05)	0.395
**Smoking**	0.56 (0.26 - 1.21)	0.14		
**Previous surgery**	0.9 (0.56 - 1.45)	0.666		
**Perianal Crohn's disease**	1.07 (0.67 - 1.7)	0.776		
**Extraintestinal Manifestations**	1.29 (0.61 - 2.72)	0.499		
**Labs**				
**Zinc deficiency**	2.9 (1.8 - 4.66)	<0.001	1.29 (0.43 - 3.83)	0.65
**High CRP**	6.2 (3.51 - 10.97)	<0.001	2.11(0.68 - 6.58)	0.199
**High FCP**	3.85 (1.54 - 9.59)	0.004	1.08 (0.34 - 3.46)	0.894
**Low Albumin**	6.92 (4.13 - 11.59)	<0.001	5.93 (1.86 - 18.87)	**0.003**
**Medications**				
**Immunomodulators**	1 (0.64 - 1.58)	0.998		
**Advanced Therapies**	0.93 (0.59 - 1.47)	0.751		
**Corticosteroids**	2.35 (1.32 - 4.18)	0.004	1.4 (0.44 - 4.44)	0.573

CRP: C-reactive protein; FCP: fecal calprotectin

Table S7: Univariate and Multivariate Analyses on Predictors of IBD-related Surgeries in Crohn's Disease.

Variable	Univariate analysis		Multivariate analysis	
	**Odds ratio (95% CI)**	**P-value**	**Odds ratio (95% CI)**	**P-value**
**Male**	1.64 (0.91 - 2.95)	0.097	2.55 (0.86 - 7.59)	0.093
**Age**	0.99 (0.97 - 1.02)	0.658		
**Disease duration**	0.96 (0.91 - 1.01)	0.103		
**Smoking**	0.63 (0.23 - 1.72)	0.368		
**Previous surgery**	0.69 (0.36 - 1.3)	0.249		
**Perianal Crohn's disease**	1.18 (0.65 - 2.14)	0.584		
**Extraintestinal Manifestations**	0.98 (0.36 - 2.65)	0.964		
**Labs**				
**Zinc deficiency**	3.57 (1.86 - 6.84)	<0.001	5.23 (1.26 - 21.65)	**0.022**
**High CRP**	9.87 (3.81 - 25.54)	<0.001	2.9 (0.71 - 11.91)	0.139
**High FCP**	3.13 (1.09 - 9.03)	0.034	0.65 (0.15 - 2.85)	0.565
**Low Albumin**	3.41 (1.87 - 6.22)	<0.001	3.3 (0.9 - 12.05)	0.071
**Medications**				
**Immunomodulators**	1.46 (0.81 - 2.63)	0.207		
**Advanced Therapies**	1.16 (0.64 - 2.1)	0.628		
**Corticosteroids**	2.37 (1.2 - 4.67)	0.013	0.76 (0.2 - 2.84)	0.682

CRP: C-reactive protein; FCP: fecal calprotectin

Table S8: Univariate and Multivariate Analyses on Predictors of IBD-related Complications in Crohn's Disease

Variable	Univariate analysis		Multivariate analysis	
	**Odds ratio (95% CI)**	**P-value**	**Odds ratio (95% CI)**	**P-value**
**Male**	1.16 (0.7 - 1.9)	0.566		
**Age**	0.99 (90.97 - 1.01)	0.553		
**Disease duration**	0.99 (0.95 - 1.03)	0.482		
**Smoking**	0.51 (0.22 - 1.16)	0.109		
**Previous surgery**	0.87 (0.52 - 1.47)	0.607		
**Perianal Crohn's disease**	1.56 (0.95 - 2.57)	0.082	1.48 (0.6 - 3.63)	0.396
**Extraintestinal Manifestations**	1.44 (0.66 - 3.14)	0.366		
		**Labs**		
**Zinc deficiency**	3.16 (1.86 - 5.36)	<0.001	3.69 (1.3 - 10.5)	**0.014**
**High CRP**	3.39 (1.92 - 5.99)	<0.001	1.39 (0.48 - 4.05)	0.545
**High FCP**	2.28 (0.97 - 5.37)	0.06	1.28 (0.41 - 3.98)	0.664
**Low Albumin**	3.3 (1.96 - 5.56)	<0.001	1.58 (0.52 - 4.8)	0.424
		**Medications**		
**Immunomodulators**	1.22 (0.75 – 2)	0.426		
**Advanced Therapies**	0.64 (0.39 - 1.05)	0.075	0.82 (0.32 - 2.08)	0.669
**Corticosteroids**	1.47 (0.79 - 2.76)	0.227		

CRP: C-reactive protein; FCP: fecal calprotectin

Table S9: Univariate and Multivariate Analyses on Predictors of IBD-related Hospitalizations in Ulcerative Colitis

Variable	Univariate analysis		Multivariate analysis	
	**Odds ratio (95% CI)**	**P-value**	**Odds ratio (95% CI)**	**P-value**
**Male**	0.54 (0.18 - 1.57)	0.255		
**Age**	0.96 (0.91 - 1.01)	0.095	0.99 (0.91 - 1.07)	0.725
**Disease duration**	0.93 (0.85 - 1.02)	0.144		
**Smoking**	1.22 (0.12 - 12.4)	0.867		
**Previous surgery**				
**Extraintestinal Manifestations**	5.93 (1.82 - 19.33)	0.003	2.43(0.33 - 17.75)	0.38
**Labs**				
**Zinc deficiency**	4.49 (1.46 - 13.8)	0.009	2.97 (0.6 - 14.69)	0.183
**High CRP**	7.76 (2.23 - 27.06)	0.001	2.36 (0.31 - 17.66)	0.404
**High FCP**	4.69 (0.94 - 23.35)	0.059	1.44 (0.17 - 12.58)	0.741
**Low Albumin**	19.54 (5.3 - 72.11)	<0.001	4.31 (0.66 - 28.31)	0.128
**Medications**				
**Immunomodulators**	0.63 (0.21 - 1.9)	0.412		
**Advanced Therapies**	3.27 (1.09 - 9.75)	0.034	2.68 (0.51 - 14.09)	0.243
**Corticosteroids**	4.16 (1.21 - 14.34)	0.024	2.38 (0.35 - 16.06)	0.375

CRP: C-reactive protein; FCP: fecal calprotectin

Table S10: Univariate and Multivariate Analyses on Predictors of IBD-related Emergency Room Visits in Ulcerative Colitis

**Variable**	**Univariate analysis**		**Multivariate analysis**	
	**Odds ratio (95% CI)**	**P-value**	**Odds ratio (95% CI)**	**P-value**
**Male**	0.46 (0.15 - 1.38)	0.166		
**Age**	0.97 (0.92 – 1.01)	0.151		
**Disease duration**	0.94 (0.86 – 1.03)	0.191		
**Smoking**	1 (**0-0)**			
**Previous surgery**	1 (**0-0)**			
**Extraintestinal Manifestations**	6.78 (2.03 - 22.6)	0.002	3.63 (0.48 - 27.28)	0.211
		**Labs**		
**Zinc deficiency**	3.89 (1.25 - 12.09)	0.019	1.17 (0.21 - 6.62)	0.86
**High CRP**	10.59 (2.69 - 41.68)	0.001	3.08 (0.42 - 22.73)	0.271
**High FCP**	11.08 (1.34 - 91.74)	0.026	3.28 (0.26 - 41.72)	0.36
**Low Albumin**	15.68 (4.31 - 56.97)	<0.001	3.02 (0.43 - 21.09)	0.265
		**Medications**		
**Immunomodulators**	0.23 (0.06 - 0.88)	0.032	0.23 (0.03 - 2.1)	0.195
**Advanced Therapies**	2.81 (0.93 - 8.5)	0.068	3.38 (0.6 - 19.06)	0.168
**Corticosteroids**	2.0 (0.54 - 7.45)	0.301		

CRP: C-reactive protein; FCP: fecal calprotectin

<u>Table S11:</u> Predictors of IBD-related Surgeries in Ulcerative Colitis

Variables		IBD-Related Surgeries		
		No<br>n (%)	Yes<br>n (%)	Chi square<br>p-value
**Age, mean (SD)**		34.9 (13.37)	26 (.)	0.51
**Disease duration, years (SD)**		8.12 (7.28)	7 (.)	0.88
**Current Smoker**		5 (100.0)	0 (0.0)	0.742
**Previous intestinal surgery**	No	84 (100.0)	0	**<0.001**
	Yes	1(50.0)	1(50	
**Extraintestinal Manifestations**	No	69 (100.0)	0	**0.043**
	Yes	16 (94.1)	1(5.88)	
**Zinc Deficiency**	No	55 (100.0)	0 (0.0)	0.18
	Yes	30 (96.8%)	1 (3.2%)	
**High CRP**	No	47 (100.0)	0(0.0)	0.215
	Yes	30 (96.8)	1 (3.2)	
**High FCP**	No	25 (100.0)	0 (0.0)	0.414
	Yes	37 (97.4)	1 (2.6)	
**Low Albumin**	No	62 (100.0)	0 (0.0)	0.069
	Yes	18 (94.7)	1 (5.3)	
**Immunomodulator**	No	47 (97.9)	1 (2.1)	0.371
	Yes	38 (100.0)	0 (0.0)	
**Advanced Therapy**	No	55 (100.0)	0 (0.0)	0.18
	Yes	30 (96.8)	1 (3.2)	
**Current corticosteroid use**	No	71 (98.6)	1 (1.4)	0.657
	Yes	14 (100.0)	0 (0.0)	

SD: standard deviation; CRP: C-reactive protein; FCP: Fecal calprotectin
